# Haptoglobin and C-Reactive Protein—Non-specific Markers for Nursery Conditions in Swine

**DOI:** 10.3389/fvets.2019.00092

**Published:** 2019-03-28

**Authors:** Isabel Hennig-Pauka, Anne Menzel, Till Robert Boehme, Horst Schierbaum, Martin Ganter, Jochen Schulz

**Affiliations:** ^1^Field Station for Epidemiology, University of Veterinary Medicine Hannover, Foundation, Bakum, Germany; ^2^University Clinic for Swine, University of Veterinary Medicine, Vienna, Austria; ^3^Drägerwerk AG & Co. KGaA, Lübeck, Germany; ^4^Möller GmbH, Diepholz, Germany; ^5^Clinic for Swine, Small Ruminants, Forensic Medicine and Ambulatory Service, University of Veterinary Medicine Hannover, Foundation, Hanover, Germany; ^6^Institute for Animal Hygiene, Animal Welfare and Farm Animal Behaviour, University of Veterinary Medicine Hannover, Foundation, Hanover, Germany

**Keywords:** climate control, ammonia, sedimentation dust, biomarker, respiratory disease

## Abstract

A quality concept for production in the pork market includes granting a good health status of pigs from birth to slaughter. This concept is a precondition for animal welfare as well as reducing antibiotic usage in farm animals. The demand for fighting bacterial antimicrobial resistance in humans, animals, and in the environment is one driving force for the development of innovative technical solutions to improve husbandry. Maintenance of a good health status in pigs depends on early detection of a disturbance in homeostasis in critical phases of life. This can be measured by non-specific biomarkers as acute phase proteins. In this project, husbandry conditions and health status in nursery pigs were monitored in an autumn and winter nursery period from weaning to the end of nursery in two compartments with 180 pigs each. It was investigated whether a slight modification in indoor climate achieved by a new ammonia sensory technology coupled with the electronic control unit of the forced ventilation system ensuring ammonia levels lower than 5 ± 3 ppm in one compartment led to a better health status in piglets in comparison to the control compartment. In the examined nursery periods in different seasons, ammonia concentrations in the experimental compartment were significantly lower than in the control compartment, thus proving the functionality and efficacy of the technical system. Production parameters as feed conversion rate and average daily weight gain were slightly improved in the experimental compartment without implementing other measures. Multifactorial analysis of variance resulted in a significant influence of season, daily quarter, and compartment on ammonia concentration. The challenge to preserve a high health status of piglets also during suboptimal outside climate in the transitional season was reflected by an increase in the acute-phase proteins haptoglobin (Hp) and C-reactive protein (CRP) in autumn compared to winter. The seasonal influence on concentrations of CRP and Hp superimposed potential influences of the climate modification. New technological concepts to reduce noxious gases and dust in the animal environment as well as emissions, which in parallel guarantee optimal temperatures also during extreme weather conditions, can be evaluated by clinical data in combination with biomarkers.

## Introduction

Weaning from the sow and moving to the nursery are two of the most stressful events in the life of a pig (1). Handling and transportation to a different physical environment, abrupt change in food source with respect to availability, composition and consistency, social hierarchical stress caused by co-mingling of different litters and exposure to new microorganisms and antigens are the most important challenges during weaning. As a stress response, corticotrophin-releasing factor (CRF) and cortisol are increased in weaned piglets by activating the hypothalamic pituitary adrenal axis (HPA) (2). At the intestinal level, CRF and the CRF receptor system play a central role in stress-induced gut dysfunction and intestinal barrier disturbances in pigs (2).

The first days after weaning of 3 to 4-week-old piglets are characterized by underfeeding, with weight loss of ~250 g because piglets have to adapt to solid feed. Full recovery of pre-weaning nutrient intake levels can be expected ~2 weeks after weaning (3). As a consequence, energy metabolism, especially with regard to glucose and protein synthesis is affected and an endocrine adjustment becomes necessary (3). Reduced brush-border digestive as well as pancreatic enzyme activities can reduce the small intestine's digestive capacity, as well as triggering the development of post-weaning diarrhea (1). Low feed intake in general predisposes to dysfunction of the intestinal barrier by a decrease in the villous length and crypt elongation, destruction of extracellular matrix protein in connective tissue and junctional proteins as a consequence of local inflammatory reactions in the gut (4–7). Inflammatory responses during weaning are also reflected by an increased expression of pro-inflammatory cytokines as IL-1ß, IL-6, and TNFα in gut tissue regulating active immune responses (8). A systemic pro-inflammatory immune response has been shown up until day 7 after weaning as well as locally in the skeletal muscle with significant alterations in muscle fibers (9). Generally, an activation of the immune system is accompanied by reduction in feed efficiency and lean tissue deposition in pigs (10, 11).

Alongside intestinal disorders, respiratory diseases influence health and welfare in nursery pigs, although effective commercial vaccines are available against most of the involved pathogens. As a consequence, the prevalence of inflammatory lung alterations found at slaughter is high and respiratory disease is a main reason for antibiotic usage in swine production (12, 13). Infectious agents such as *Mycoplasma* (*M*.) *hyopneumoniae, Actinobacillus pleuropneumoniae* (*A.pp*.), Porcine Reproductive and Respiratory Syndrome Virus (PRRSV) and influenza virus, as well as climatic factors such as dust and hazardous gases, have a synergistic effect in disease pathogenesis (14, 15). The awareness of the impact of non-infectious factors for disease pathogenesis is increasing. Suboptimal husbandry conditions triggering the development of disease, which then has to be treated with antimicrobial substances as a consequence, are one reason for voicing criticism concerning industrial pork production. One aim of the global one-health initiative to fight antibacterial resistance is to reduce antibiotic usage in livestock as much as possible (16).

The ongoing challenge for farmers and veterinarians is to retain productivity and health in swine herds in spite of colonization of pigs with potential pathogenic microorganisms. Early recognition and prevention of any trigger factors causing stress, inflammation or leading to activation of pathogens, is indispensable. Any appropriate markers for maintaining homeostasis aid in assessing animal health and welfare. In this context, acute-phase proteins (APP) as infection and inflammation markers, but also as stress indicators for farm animals can be useful (17, 18).

There are a great number of experimental and field studies confirming the use of APP as indicators for various infectious and inflammatory diseases, but only a few studies used APP to diagnose stress in farm animals. Various studies suggest that relationships between different biomarkers and their precursors and combinations thereof allow a more precise definition of pathological processes than is the case with single markers (19–22). CRP and SAA are the first line rapid reacting APPs increasing within hours. In pigs, the second line acute phase protein Hp is increased later with elevated concentrations lasting for up to 2 weeks (17). Cut-off values for single pathogens have been established for most relevant APP in swine (21). It was found that pigMAP was not elicited after infection with PRRSV (23), while Hp and CRP were used successfully to assess disease caused by PRRSV (21, 24). After PRRSV infection, serum, saliva, and meat juice were all found to be appropriate sample types for determining Hp and CRP (24). Time-resolved immunofluorometry has been developed successfully to determine both parameters in parallel in meat juice for detecting diseased animals (25). The extent of lung alterations due to *M. hyopneumoniae* was found to be correlated with Hp but to a lesser extent with CRP (26). In pigs experimentally infected with *A.pp*., already in the early stage 68 h after infection, an increase in CRP was observed (27). Four days after infection, CRP, HP, and ceruloplasmin were elevated but returned to base-line levels up until 3 weeks after infection (28). APP concentrations in general seem to be lower in viral than in bacterial infections (23), but Hp was assessed to be a more general disease marker (26).

In swine, APP were used in experimental infection to monitor immune reactions (28) or for diagnosing herd health status to monitor a change in homeostasis prior to the onset of clinical disease, trauma and stress response (21). Within minutes after a harmful influence on tissue cells (fibroblasts, endothelial cells, macrophages), secreted cytokines serve as messengers between the local body sites and the hepatocytes, where APP are produced within hours (17). Type 1 APP as α1-acid glycoprotein, serum amyloid A, Hp and CRP are induced by IL1-type cytokines (e.g., IL-1α, IL-1β, TNF α, TNFβ) and IL-6, while type 2 APP as fibrinogen, α1-antitrypsin and ceruloplasmin are induced by IL6-type cytokines (17). The connection between acute-phase response and non-inflammatory and psychological stress response is the activation of the HPA- and the sympatho-adrenal axis by releasing glucocorticoids and catecholamines. Both stimulate the production of pro-inflammatory cytokines such as IL-1, IL-6, and TNF-α by immune cells as a stress response, which elicits the acute-phase response (29). Glucocorticoids and pro-inflammatory cytokines are potentially direct stimulators of the acute-phase response (30, 31). A variety of salivary stress biomarkers has been identified for pigs including salivary cortisol, chromogranine A, immunoglobuline A, and testosterone (32). It can therefore be postulated that two major stress pathways are induced by weaning with a subsequent production of hepatic APPs.

An influence of rearing conditions on serum Hp levels has been previously shown. Nonetheless, they can also be linked to the infection dynamic in the herd. Pigs reared in pens with more space and solid partitions between pens showed lower Hp levels but, in parallel, had fewer lung lesions caused by *M. hyopneumoniae* (26). Several APP including Hp and CRP changed significantly when pigs were exposed to different housing conditions (33). Mixing animals was seen as an important stressor leading to increased APP concentrations (34) as were a change in feeding technique (35) and long distance transport (36–38). In healthy pigs after weaning, a significant increase in Hp-, CRP-, pigMAP-, and SAA-concentrations was found (39). As stress leads to muscle protein degradation provoked by elevated cortisol levels, newly synthetized APP from amino acids in the liver might be one reason for elevated APP after weaning (39, 40). The acute-phase response elicited by the weaning process might also be a result of the increased expression of pro-inflammatory cytokines in the gut (8). Systematic studies in swine using Hp- and CRP-concentrations for assessing nursery conditions are rare.

The aim of the present study was to assess the effects of a new climate control device for ammonia reduction in the air on production and clinical parameters as well as serum Hp- and CRP-concentrations in nursery pigs. The influence of abiotic factors such as ammonia and dust under field conditions was assessed by combining the given parameters.

The hypotheses of the study were that eliminating NH_3_ as an abiotic factor triggering respiratory disease in swine would lead to (i) improvement in animal health reflected in fewer skin lesions, coughing and diarrhea as the consequence of less stress and less animal-animal interaction, (ii) improvement in average daily weight gains and feed conversion rates, (iii) fewer inflammatory reactions in the respiratory tract reflected in a decrease in APP concentrations.

## Materials and Methods

### Study Design

**Figure 1:**
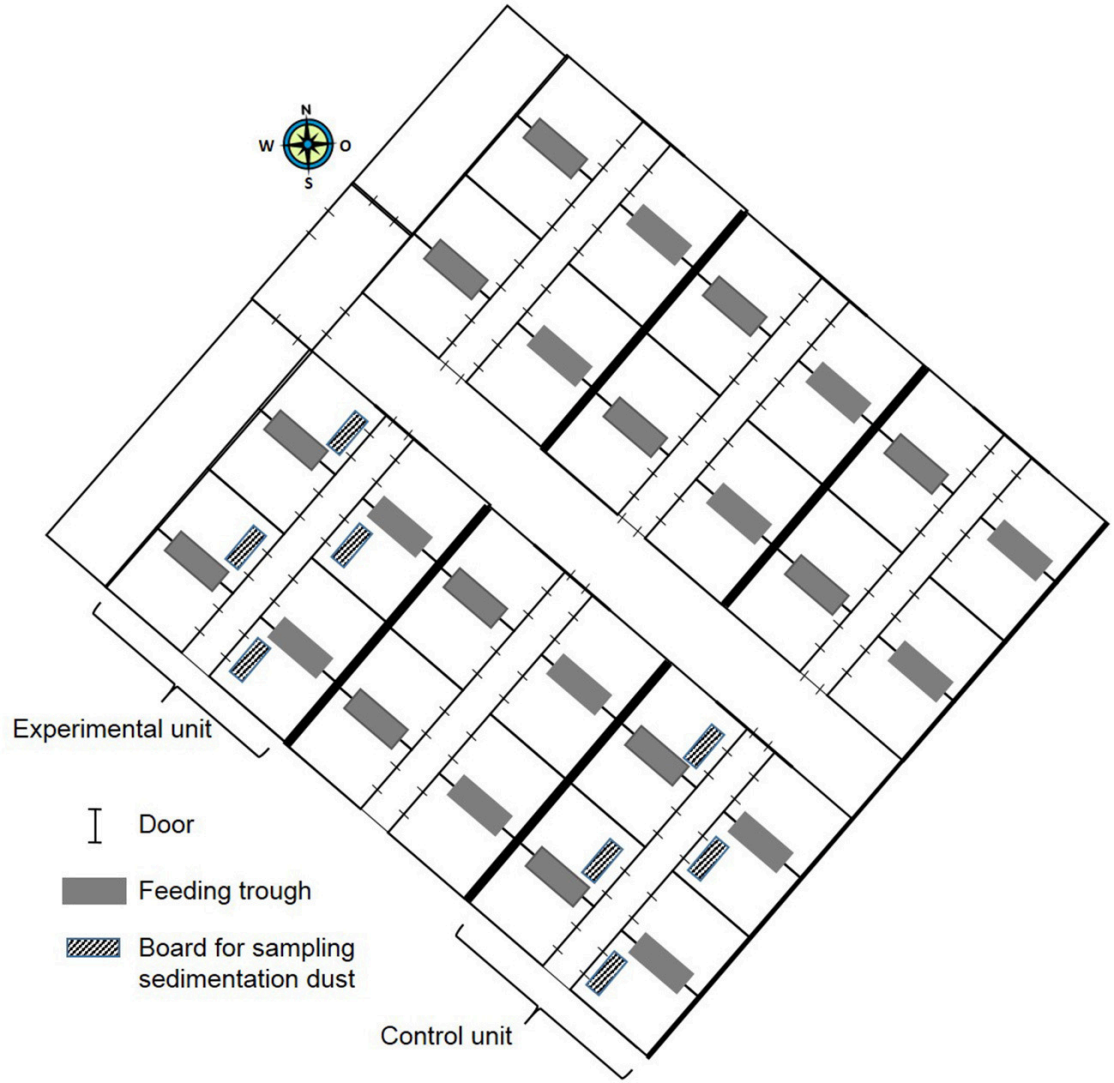
Schematic map of nursery building containing six compartments identical in construction with space for 180 pigs each.

**Figure 2:**
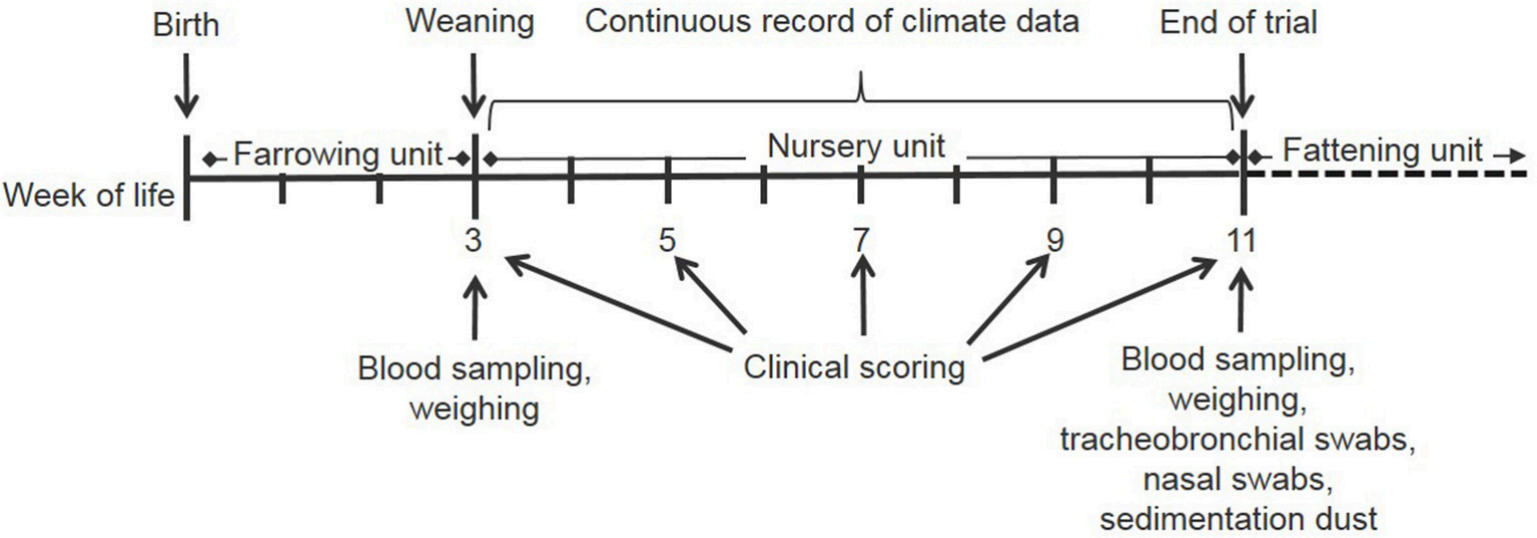
Time schedule of the field study during one nursery period starting on the day of weaning and ending at the day of moving the pigs to the fattening unit.

In this experimental field study, the effect of ammonia control in the air on the health and performance of nursery pigs was assessed in two experiments carried out in the autumn and winter. The study was performed on a farrow-to finishing farm with 200 sows in North Western Germany. Sows were allocated to groups of 40–44 animals farrowed group-wise in farrowing crates at 4-week intervals. After a 3-week suckling period, piglets were separated from the sows and transported to the nursery stable ~50 m away. In total, ~540 piglets were weaned every 4 weeks and moved litter-wise to three out of six nursery compartments each housing 180 piglets. For one experiment, 360 piglets were divided into two groups of 180 piglets and moved to two separate nursery compartments equipped with the respective sensor and ventilation technique as well as with hanging boards of defined size for sampling sedimentation dust (Figure 1). The experimental set-up is depicted in Figure 2. Piglets were marked with ear tags and weighed individually at the day of weaning after being transported to the nursery compartments. Paired blood samples were taken from 10 piglets from all pens within each compartment at the day of weaning as well as 7 weeks later at the end of the nursery period. At the end of the nursery period, in parallel, tracheobronchial and nasal swabs were taken from the same pigs. At the day of weaning, and at 2-week intervals, animals were inspected by a veterinarian five times during the nursery period for clinical symptoms, especially for coughing and sneezing, diarrhea, lameness, and skin lesions (Figure 2). The dirtiness of animals and pens were evaluated. Clinical findings were translated to quantitative scores. At the end of the nursery period, the sedimentation dust was harvested for further diagnostics. All pigs were weighed again to calculate the average daily weight gains (ADG) and the feed conversion rate (FCR). Feed consumption during the nursery period was recorded by weighing all feed filled in the four automatic feeders in each compartment.

All piglets were housed in accordance with the German welfare regulations. The European Convention regulations concerning the protection of vertebrate animals and those of the Directive of the European Convention for the Protection of Vertebrae Animals used for Experimental and Other Scientific Purposes (European Treaty Series, nos. 123 and 170) within the legislation on the protection of animals used for scientific purposes were followed wherever possible. The European Parliament and Council Directive 2010/63/EU of 22 September 2010 on the protection of animals used for scientific purposes was therefore also followed with the exception of the provision of bedding and rooting material and solid flooring. As mentioned in the European Treaty Series, no. 123, animals used for experiments performed on commercial farms, which require commercial conditions, had to be kept under those housing conditions stipulated in the European Convention for the Protection of Animals Kept for Farming Purposes (ETS No. 87). These conditions were fulfilled in this study. The study was approved by the Lower Saxony State Office for Consumer Protection and Food Safety (LAVES) and in accordance with the requirements of the national animal welfare law (Approval Number: 33.19-42502-05-16A041). Precautions aimed at avoiding unnecessary suffering of the animals were taken at all stages of the experiment.

### Husbandry Conditions on Farm and Climate Control

The trial was performed in two outer compartments of the six nursery compartments located on one side of the central corridor with space for 180 piglets each. Both nursery compartments were confined by an outer as well as an inner wall (Figure 1). Each of the nursery compartments contained eight pens with space for 22 piglets, with four pens at each side of the feeding corridor (Figure 1). Piglets belonging to one litter were rehoused in one pen in the nursery compartment. In each pen, two litters were mixed. This litter-wise weaning management had been implemented on the farm to reduce stress and transmission of pathogens, which might be caused by mixing piglets from various litters. In two pens, one automatic feeder for *ad libitum* grid feeding was installed (Figure 1). Two water supplies at the automatic feeders were installed in each pen as well as three separate nipple drinkers. After the piglets had been moved to the nursery compartments at the day of weaning, the room temperature was increased to 32°C.

The compartments were equipped with a diffuse ceiling ventilation and an underfloor suction system. The stable had a fresh air duct in the pitched roof. The fresh air trickled through the perforated ceiling into the compartments. The exhaust air was sucked out through the slatted floor and taken away through an exhaust air duct above the corridor.

Both compartments were equipped with a conventional ventilation system (Möller GmbH, Diepholz, Germany). Two process-controlled digital voltage regulators (microprocessor control type DR 1-D, Möller GmbH) were installed in the central corridor, connected to the sensors in the respective nursery compartment as well as to the ventilation system via a 0–10 V interface, which is widely used in agriculture. The 0–10 V signal was used to display the measured value, with 0 V equalling no or the lowest measurable value and 10 V indicating the upper end of the measuring range. The two compartments were equipped with a temperature, relative humidity, carbon dioxide and ammonia sensor. All sensors were installed ~ 1 m above the floor to ensure that they were placed close to the animals but still out of their reach. All sensors measured continuously and were connected to the ventilation system via the climate control facility during the entire experiment. The temperature sensor covered a temperature range of −20 to 40°C (Möller GmbH). The relative humidity sensor (E+E Elektronik Ges.m.b.H., Engerwitzdorf, Austria) measured percentage humidity in the range of 0 to 100 RH. The CO_2_ measuring device (Vaisala Oyj, Helsinki, Finland) had a measuring range of 0 to 10 000 ppm. Measuring the ammonia concentration in the air was enabled by installing the first ammonia sensor developed for continuous measurements in livestock houses (Polytron C300, Drägerwerk AG & Co. KGaA, Lübeck, Germany). The measuring range of this sensor is 0 to 100 ppm with an accuracy of ± 1.5 ppm or ± 10% of the measured value.

The climate control facility was able to regulate indoor climate for each of the four measured parameters, by setting respective minimum and maximum values and controlling the ventilation accordingly. As standard, the guiding parameter for the climate inside the stable was room temperature, so that ideal rearing conditions depending on weight and age of the piglets were guaranteed.

As in most conventional nursery compartments, ventilation in the control compartment was simply regulated based on the temperature. The system was adjusted to stabilize room temperature according to the manually defined climate curve during the nursery period. In the experimental compartment, the ventilation system was activated when the ammonia concentration exceeded 5 ppm over a control range of 3 ppm. Ventilation was reduced at the end of the control range or when the measured temperature fell below the defined range.

### Examination of Animals and Sampling

#### Average Daily Weight Gain and Feed Conversion Rate

Approximately 360 pigs aged 3 weeks were weighed and marked with ear tags individually at the day of weaning after being transported to the cleaned and disinfected nursery compartments. After a time period of 7 weeks, the pigs were weighed again and brought to the fattening unit (Figure 2). The body weight of pigs at the end of the study at this time-point was 27.8 ± 4.6 kg (mean±standard deviation). The average daily weight gain (ADG) during nursery was calculated. Piglets in one nursery period in the autumn and one in the winter were weighed individually.

The farmer observed the piglets daily. Feed filled into the automatic feeders was weighed by the farmer, so that feed consumption was recorded for each automatic feeder (one for every two pens). Feed conversion rates (FCR) were calculated retrospectively based on the ADG of pigs in the two pens allocated to the respective automatic feeder.

#### Clinical Examination of Pigs and Assessment of Environment

Clinical examination by a veterinarian was performed at five time-points: at the day of weaning, which was the start of the nursery period at the age of 3 weeks and at 2-week intervals up until the end of the nursery period (Figure 2). At each examination day, the coughing index was determined in each pen as described elsewhere (41). Briefly, coughing was recorded during a 3-min observation period per pen and divided by the number of animals observed and the minutes to calculate the percentage of coughing of the animals per minute. In addition, the percentages of animals with skin lesions and diarrhea were recorded per pen. In parallel to the clinical examination, each pen was assessed regarding the degree of dirtiness: score 1: < 25% piglets dirty up to knee/elbow, score 2: >25% piglets dirty up to knee/elbow, score 3: >25% piglets dirty all over.

#### Sampling of Animals, Determining CRP and Hp and Microbiological Diagnostics

In the autumn and winter nursery periods, nasal swabs, and blood samples were taken from 10 piglets as paired samples at the day of weaning and at the last day of nursery. Serum samples were analyzed for CRP and Hp. CRP was measured by a commercial ELISA (Phase Porcine CRP Assay, Tridelta Development Limited, Maynooth, Ireland) and Hp was determined in serum using a colorimetric method (Tridelta Phase Haptoglobin Assay, Tridelta Development Limited) in accordance with the manufacturer's instructions. In addition, tracheobronchial fluid (TBF) was collected at the last day of nursery. Briefly, in fixated pigs, a tube was inserted into the trachea without visual control and retracted immediately so that undiluted tracheobronchial fluid could be harvested from the tube due to capillary forces (42). TBF was examined by PCR for *M. hyopneumoniae* following a routine diagnostic method. Nasal swabs were examined for methicillin-resistant *Staphylococcus aureus* (MRSA) by culture.

In serum samples from the autumn period, antibodies against *M. hyopneumoniae, A.pp.*, PRRRSV and influenza virus, as the major pathogens causing respiratory disorders in pigs, were examined by commercial ELISA.

#### Sampling and Analysis of Sedimentation Dust

Sedimentation dust was sampled in three nursery periods in the summer, autumn and winter. In each compartment, four boards measuring 0.4 × 0.6 m^2^, covered with aluminum foil, were hung horizontally at the height of 1.90 m at the day of weaning. Sedimentation dust was harvested from these boards after the end of the 7-week-nursery period by weighing the dust-covered foils and then brushing the dust into sterile vials. For determining the total amount of viable and culturable bacteria in the sedimentation dust, 100 mg of sedimentation dust was warmed up in a water bath for 30 min at 25°C in 10 mL phosphate-buffered saline (PBS) with 0.01% Tween20 and shaken at 120 rpm. Subsequently, the suspension was vortexed for five min at 2,400 rpm. Aliquots (0.1 mL) of serial dilutions were plated in triplicate on blood agar plates (Oxoid Limited, Hampshire, United Kingdom) and incubated at 36°C for 48 h. Colonies were counted and the total amount of viable bacteria per gram dust (CFU/gram) was calculated. For detecting MRSA, 0.5 g sedimentation dust was dissolved in 50 mL PBS with 0.01% Tween20. Qualitative and quantitative analyses were conducted in accordance with Friese et al. (43). To confirm suspected MRSA isolates, the S*taphylococcus aureus* specific *nuc* gene and the *mecA* gene were amplified as described by Clauss et al. (44).

For determining dust-bound ammonia, 20 mg sedimentation dust were solubilized in 10 mL A test acidified with 10 μL 10% peracetic acid to adjust the pH-value to 5.5–6.5. After centrifuging for 10 min at 10 000 g the clear supernatant was used for ammonia quantification using the LT-Sys® commercial test kit (Labor+Technik, Berlin, Germany). NH_3_ was photometrically determined by measuring extinctions of standard and samples after a 1- and 3-min reaction time at 25°C following the manufacturer's instructions. Measurements were based on the chemical reaction of α-ketoglutarat + NH4^+^ +NADH to L-glutamate + NAD^+^ + H_2_O by adding glutamate dehydrogenase (GLDH). Optical densities were measured at wavelength 334 nm.

### Statistical Evaluation

For statistically evaluating climate data, the mean values of 6 h from the minutely recorded data, i.e., daily quarter values, were calculated. The daily quarter values were determined based on the main circadian rhythms of the pigs under husbandry conditions (45) as follows: 06:00–12:00 (behavior according to comfort, feed intake, resting, activity), 12:00–18:00 (behavior according to comfort, feed intake, resting, activity), 18:00–00:00 (resting), 00:00–06:00 (mainly resting).

For time course inspection during the entire nursery period, mean values of the respective days were taken into account. Data were summarized in the Excel program (Microsoft® Office Excel 2016, Redmond, Washington, USA) and analyzed using the statistical program SAS® (SAS-Modul Enterprise Guide, SAS Institute Inc., Cary, NC, USA). For evaluating climate data, three influencing fixed effects: nursery period (season), quarter of the day and the compartment (control vs. experimental) were included in the model in a multifactorial variance analysis. The clinical parameters: coughing index, percentage of animals with diarrhea and skin lesions as well as pen dirtiness and CRP- and Hp-concentrations were analyzed by a multifactorial variance analysis. In addition, nursery periods as well as both compartments within one nursery period were analyzed separately by pair-wise statistical comparison of control and experimental compartment data using the non-parametrical Wilcoxon Test. Pair-wise comparisons of Hp and CRP between the beginning and end of the nursery period were compared using the Signed Rank Test. Spearman correlation coefficients were evaluated for all quantitative parameters. Frequencies between both compartments were compared using the Chi-Square Test.

## Results

### Climate Data

**Figure 3:**
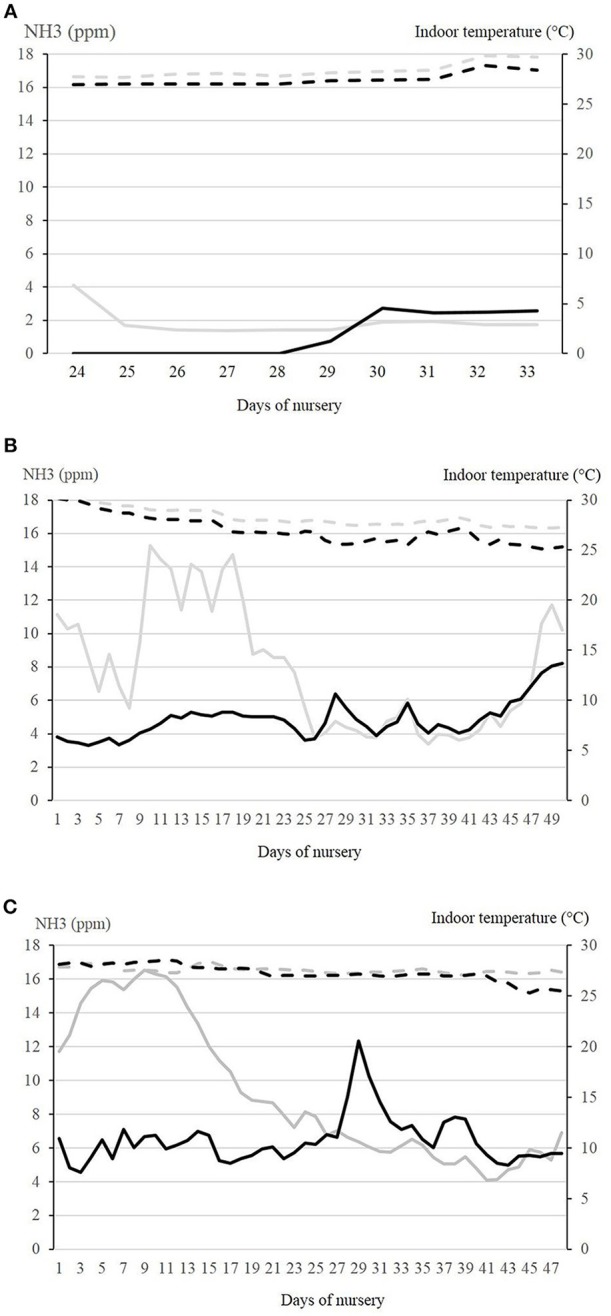
Courses of ammonia concentrations and indoor temperature are shown for the different seasons at the respective days of nursery. In summer **(A)**, measurements were performed between days 24 and 33. Environmental temperature in this period was between 13.6 and 12.9°C in July 2016. In autumn **(B)** and winter **(C)**, measurements were recorded for the whole nursery period. In autumn, environmental temperature was between −0.4 and +25.2°C (Sept.–Nov.). In winter, environmental temperature was between−5.3 and +7.9°C (Jan.–March). NH_3_ concentration (ppm) is depicted by the continuous lines in the control compartment (gray) and the experimental compartment (black). Indoor temperature (°C) is depicted by the broken lines in the control compartment (gray) and the experimental compartment (black).

The courses of mean ammonia concentrations in the air and indoor temperatures in the control and experimental compartment in the three seasons are shown in Figures 3A–C. In the control compartment in winter, the ammonia concentration was positively correlated with room temperature, relative humidity, and percentage of heating power, while it was negatively correlated with the CO_2_-concentration, the outer temperature and ventilation rate. In the experimental compartment, the ammonia concentration was not correlated with the outer temperature. In addition, the higher the heating rate in this compartment, the lower the ammonia concentration was.

Multifactorial analysis of variance resulted in a significant influence of season, daily quarter values and compartment on ammonia concentration, while interactive effects between the fixed effects were of lower impact. In all examined nursery periods in the different seasons, the ammonia concentrations in the experimental compartment were significantly lower than in the control compartment.

### Clinical and Laboratory Diagnostic Findings

#### Average Daily Weight Gain and Feed Conversion Rate

Overall, comparison of data in both compartments independent of season resulted in significantly higher ADG (*p* = 0.0046) in the experimental compartment. ADG was higher in the experimental compartment in all seasons with improved or similar FCR. This finding was significant in the autumn with improvement in the ADG (*p* = 0.05) as well as FCR (*p* = 0.05). The chosen statistical model with the fixed effects of the season and compartment explained the differences in ADG to 27% showing a significant influence of the compartment and to 74%, showing a significant influence of the season. Worst FCR was found in summer and best in winter.

#### Clinical Findings

The descriptive statistical data of clinical findings are shown in Table 1 for the autumn and winter nursery period. The health status of the animals was assessed as good at all examination times and therefore it was unnecessary to treat the groups of animals. Most clinical parameters did not differ significantly between the autumn and winter period, with the exception of mild diarrhea in the first 2 weeks after weaning, which was significantly more severe in winter (*p* = 0.009).

**Table 1:** Clinical and laboratory diagnostic findings in nursery compartments.

	**Control compartment**			**Experimental compartment with additional climate control**			***P*^b^**
	***n***	**Median**	**Range**	***n***	**Median**	**Range**	
	**Autumn (september-november)**						
Pen-wise average daily weight gain (g)	8	397	364–456	8	446	387–492	**0.05**
Feed conversion rate per feeding trough (kg/kg)	4	1.67	1.61–1.72	4	1.54	1.42–1.61	**0.03**
Sedimentation dust per board (g/m^2^/24 h)	4	2.8	2.4–2.9	4	2.9	2.1–4.2	0.89
Total amount of bacteria in sedimentation dust (CFU/g)	4	1.3 × 10^8^	2.1 × 10^7^-2.6 × 10^8^	4	2.5 × 10^8^	1.1 × 10^8^-3.3 × 10^8^	0.31
MRSA in sedimentation dust (CFU/g)	4	4625	334–11018	4	4783	3297–5993	1
Dust-bound NH_3_ (mg/g)	4	2.83	2.76–3.08	4	2.42	2.38–2.59	**0.03**
Coughing index^a^ end of nursery per pen	8	0	0–4.8	8	0	0–4.6	0.94
Haptoglobin (mg/mL) end of nursery	10	0.98	0.04–2.50	10	0.47	0.18–0.95	0.16
CRP (μg/mL) end of nursery	10	564	349–1600	10	474	166–652	0.09
	**Winter (january-march)**						
Pen-wise average daily weight gain (g)	8	425	399–477	8	449	394–568	0.23
Feed conversion rate per feeding trough (kg/kg)	4	1.48	1.35–1.65	4	1.50	1.47–1.57	0.67
Sedimentation dust per board (g/m^2^/24h)	4	2.5	2.2–3.3	4	2.9	2.4–3.2	0.67
Total bacteria in sedimentaion dust (CFU/g)	4	2.0 × 10^8^	1.1 × 10^8^-3.3 × 10^8^	4	1.2 × 10^8^	9.8 × 10^7^-1.6 × 10^8^	0.67
MRSA in sedimentaion dust (CFU/g)	4	5060	0–2662	4	1495	995–1988	0.47
Dust-bound NH_3_ (mg/g)	4	2.19	2.10–2.69	4	2.02	1.87–2.28	0.19
Coughing index^a^ end of nursery per pen	8	0	0	8	0	0	1
Haptoglobin (mg/mL) end of nursery	10	0.14	0.06–1.43	10	0.13	0.07–0.32	1
CRP (μg/mL) end of nursery	10	568	168–814	10	634	283–858	0.16

*MRSA methicillin-resistant Staphylococcus aureus*,

a *Coughing index: percentage of coughing pigs per minute*,

b *Probability value, level of significance p ≤ 0.05, pair-wise comparison by two-sided, non-parametric Wilcoxon-Two-Sample Test. Probability values in bold depict significant differences between compartments*.

In the winter, a higher coughing index was observed in the experimental compartment in the first days after weaning compared to the control compartment. Overall, the coughing index was significantly influenced by the compartment according to the chosen statistical model (*p* = 0.05). The factor point of time of examination was not included in the statistical model for evaluating the coughing index because no significance was found for this effect in a first statistical approach with three fixed factors (season, compartment, point of time of examination). No significant influence of the compartment, season and point of time of examination was found for the incidence of diarrhea, while skin lesions and dirtiness of pens were significantly influenced by the factors season and point of time of examination (<0.0001), but not by the compartment.

Pair-wise comparison within the nursery period in the autumn resulted in a tendency toward more coughing animals (*p* = 0.08) and more skin lesions (*p* = 0.015) in the experimental compartment at the beginning of the observation period than in the control compartment. At the second point of time of examination, still more coughing animals were found in the experimental compartment (*p* = 0.03), while no differences were found in the following points of time of examination. The same observation was made in winter, when at the beginning of the nursery period, piglets in the experimental compartment showed a higher coughing index (*p* = 0.01) and more skin lesions (*p* = 0.0009). A higher coughing index was still observed at the second point of time of examination in the experimental compartment (*p* = 0.0003), while at this time period in the control compartment, a higher percentage of pigs showed diarrhea (*p* = 0.05). At subsequent examinations during the nursery period, differences between both compartments were no longer detectable.

#### Laboratory Diagnostic Findings

Descriptive statistical data of laboratory diagnostic findings are shown in Table 1 for the autumn and winter nursery period. Independently of the compartment, in the autumn, the percentage of seroreagents changed from the day of weaning to the end of the nursery period: *A.pp*.: from 95 to 50%; PRRSV: from 60 to 10%; influenza virus: from 95 to 5%; *M. hyopneumoniae*: from 5% to 10%, respectively. Antibodies at the day of weaning reflected passively acquired maternal colostral antibodies. Antibodies against *A.pp*. ApxIV (*p* < 0.0001), PRRSV (*p* = 0.03), and influenza virus (*p* < 0.0001) declined during the nursery period, while antibody levels against *M. hyopneumoniae* remained at the same level. TBF samples taken at the end of the nursery period and tested by PCR for *M. hyopneumoniae* were always negative. In the autumn, 90% of all pigs were positive for MRSA in their nasal cavities irrespective of the compartment. In the winter, in the experimental compartment 90% of the sampled pigs and in the control compartment only 20% were positive for MRSA in their nose. This difference was significant (*p* = 0.002).

Overall, APP concentrations measured in this study in the autumn and winter nursery periods were in the range of 0.04–2.85 mg/mL (median 0.1 mg/mL) Hp and 4.8–1,600 μg/mL (median 406 μg/mL) CRP. If age-independent cut-offs for non-specific inflammation of Hp of 0.85 mg/mL and CRP of 19.39 μg/mL serum were taken as a basis (21), for CRP, all pigs had values exceeding the cut-off. In the autumn, in the control compartment 50% of animals and in the experimental compartment 20% of the pigs had values exceeding the cut-off for Hp. This difference was not significant. In the winter, only one pig showed an elevated Hp value.

Age-dependent upper reference limits for Hp have been published for pigs aged four (2.6 mg/mL) and 12 (3.37 mg/mL) weeks, respectively (46). In our study, these values were only exceeded in one pig at the day of weaning. According to age-related CRP-concentrations determined by Pomorska-Mol et al. (39), values in healthy pigs of the age of 3 weeks should not exceed 25 μg/mL and those of the age of 10 weeks should not exceed 40 μg/mL (39). In our present study, only two pigs showed lower concentrations at the day of weaning.

**Figure 4:**
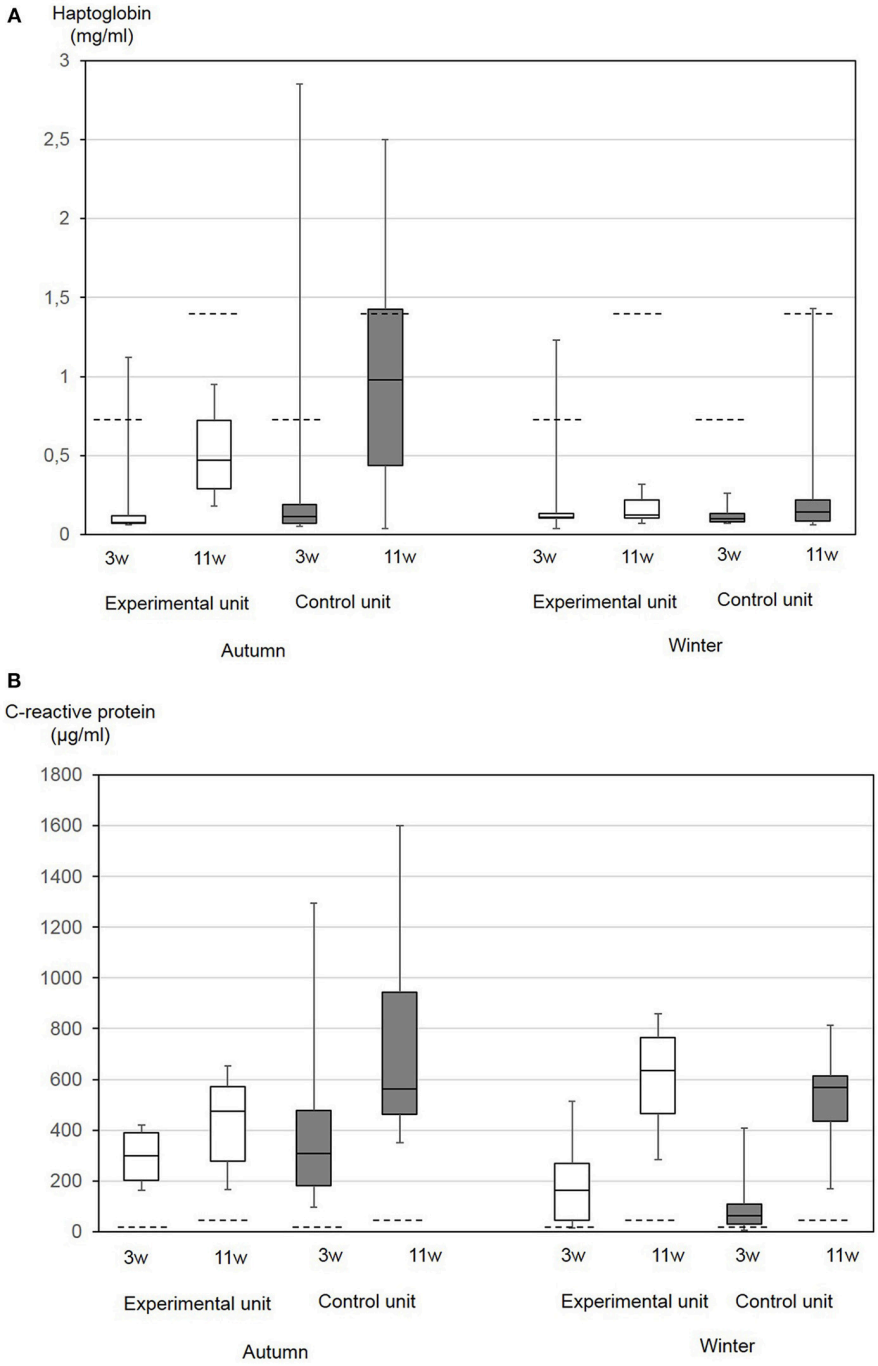
Haptoglobin **(A)** and CRP **(B)** serum concentrations in 10 nursery pigs at the day of weaning of the age of 3 weeks (3w) and at the end of the nursery period at the age of 11 weeks (11w). Piglets were either kept under experimental conditions with low ammonia concentrations in air (experimental compartment) or in a conventional nursery unit (control compartment). The trial was performed in autumn and in winter. The boxes represent the 50% between 25 and 75% quartiles. The line inside the box indicates the median. The top and bottom lines denote maximum and minimum values. Broken lines indicate previously published reference values for the respective age group: Upper reference value for haptoglobin in piglets 4 weeks of age 2.6 mg/mL and 12 weeks of age 3.37 mg/mL (46). Mean and standard deviation of CRP in piglets 3 weeks of age 18+7 μg/mL and 10 weeks of age 25+15 μg/mL (39).

Generally, between the day of weaning and the end of the nursery period, a significant increase in both parameters was found (Figures 4A,B). The differences between the first and second Hp concentrations were significantly correlated to the total bacterial cell counts in the sedimentation dust (*p* = 0.04), while the differences between the first and second CRP concentrations were significantly correlated with the dust-bound ammonia in the sedimentation dust (*p* = 0.004).

At the day of weaning, CRP values differed between seasons with significantly higher values in the autumn (*p* = 0.0006), while Hp did not differ. At the end of the nursery period, Hp concentrations differed between the seasons with higher values in the autumn (*p* = 0.0003). Pair-wise comparisons of data from the two compartments did not result in significant differences. Multifactorial analysis of variance resulted in a significant influence of the season on Hp (*p* = 0.0014) and CRP (*p* = 0.03). The CRP at the day of weaning was significantly correlated with the FCR (*p* = 0.01), diarrhea (*p* = 0.01), and skin lesions (*p* = 0.05).

#### Bacteria and Dust-Bound Ammonia in Sedimentation Dust

No significant difference in the amount of sedimentation dust and total bacterial load (CFU) in the dust was found between the seasons. Concentrations of dust-bound ammonia (*p* = 0.004) as well as MRSA (*p* = 0.01) were higher in the autumn (Table 1). The concentration of dust-bound ammonia was significantly reduced in the experimental compartment compared to the control compartment, with lower concentrations in the autumn (*p* = 0.03). Overall, the FCR was correlated with the concentration of dust-bound ammonia (*p* = 0.02). Dust-bound ammonia, CFU, and MRSA were significantly influenced by the season with lowest values in the summer.

## Discussion

Climate factors, especially dust, ammonia, draft, and high differences in minimal and maximal daily temperatures, have a high impact on health and well-being in nursery pigs. Climate and other stressors are not easily quantifiable under practical conditions and their impact on homeostasis is difficult to assess. Most assumptions are based on empirical reports. It was demonstrated in this study that dependent on outside temperature, climatic changes occurred in the stable due to inheritance of the regulatory mechanisms of the ventilation system as well as energy saving measures. In the control compartment in winter, the ammonia concentration was highly dependent on room temperature, relative humidity, and percentage of heating power. The negative correlation with the CO_2_-concentration, the outside temperature and ventilation rate indicated that in winter a low outer temperature is connected with higher heating power, lower ventilation rates and as a consequence also higher ammonia concentrations. In the experimental compartment, ammonia was the additional regulatory factor influencing the ventilation rate, so that ammonia concentration was not correlated with the outside temperature anymore. The higher the heating rate, the lower the ammonia concentration was. A most problematic situation for porcine health was demonstrated by conditions in autumn and winter, when lower ventilation rates are recommended as they provide the optimal temperature but lead to higher noxious gases and more dust on conventional farms. This drawback of reduced ventilation to keep warmth inside the building was eliminated in the experimental compartment in which the ventilation rate remained high at the cost of additional heating. In every nursery period/season, ammonia concentration in the experimental compartment was significantly lower than in the control compartment, thus proving the functionality and efficacy of the technical system.

One draw-back of this field study was that the compartments for the control and experimental group could not be changed to perform the experiment again in the changed rooms and avoid any bias caused by the construction and location of the building. The installation of the experimental climate control device was a structural measure within the respective compartment. As shown in Figure 1, the nursery stable consisted of three compartments of identical shape and equipment, so that potential influences from the geographical direction of the building might be neglected. For the field study, only the outer compartments equally confined by an outer and an inner wall were selected, so that potential influences from the outside temperature impacting temperature of the outer wall might have been the same in both compartments. Applied research to assess the impact of an innovative ventilation system should be performed under the same housing conditions in which the feasibility of the new approach has to be proven.

In general, levels of ammonia as the most important gaseous hazard in conventional housing systems, depend on the floor design, slurry system and air flow directions (47). Although in Germany the upper tolerable limit for ammonia is 20 ppm, already lower concentrations of 5 ppm can impact health and favor coinfections in swine husbandry (48). Specific effects of ammonia have been analyzed in cell-culture experiments at a biochemical level (49–52). In behavioral studies in pigs, it was shown that pigs already avoid low ammonia levels if they are given the choice to do so (53). For this reason, the regulatory pre-setting of the sensory system in the experimental compartment was fixed to 5+3 ppm in this study, so that low ammonia levels during the entire nursery period were guaranteed.

In this study, sedimentation dust was sampled at a height of 1.90 m, suggesting that the sampled dust was previously airborne dust. With 2.1–4.2 g/m^2^ a day, the amount of sedimentation dust was comparable to 2.5 g/m^2^ a day measured in a fattening unit (54). Most of the dust particles in pig barns are below the aerodynamic diameter of 1 μm (54, 55). However, the size spectra of airborne dust particles in pig barns indicate that particles sizes belong to the so-called inhalable dust fraction (55, 56). Taking the median mass diameter of particles from pig barns and the deposition characteristics of pig lungs into account, we can assume that most of the inhalable dust mass deposit was in the upper respiratory tract (55, 57). Thus, a lesser mass, but more particles reach the tracheobronchial and alveolar regions. Dust in swine confinements consist of particles from feed, skin, and feces carrying staphylococci and streptococci to a high percentage, and molds, yeasts, and enterobacteriaceae to a low percentage (58, 59). Particles regardless of biological compounds can trigger inflammatory responses by hypertrophy and agglutination of cilia (60–62). Some infectious agents might have a growth advantage in dust.It was shown that in dust-exposed pigs, CD163 expression on macrophages is higher, which is a receptor for PRRSV, so that virus entry might be facilitated (63). Dust particles are carriers of *Streptococcus suis* and livestock-associated MRSA, which are both pathogens with zoonotic potential (64, 65). In this study, the amount of sedimentation dust and total bacterial load within dust did not differ between the seasons, but concentrations of dust-bound ammonia and MRSA were higher during the autumn. Furthermore, MRSA was found in all sedimentation dust samples and in the nose of 90% of the pigs in the autumn and in 55% of the pigs during the winter. In the winter, detection rates in the control compartment with lower ventilation rates were significantly lower. One explanation for this is that sedimentation dust movement was lower due to lower air velocity associated with a lower ventilation rate compared to the experimental compartment. MRSA in sedimentation dust might be the main source for nasal MRSA colonization. For this reason, it proved impossible to reduce the number of MRSA positive carrier animals by increased ventilation rates.

In addition, we measured dust-bound ammonia, which might be of even higher importance for respiratory health, when dust particles have an impact on the respiratory epithelium and bound ammonia is solubilized in the epithelial lining fluid, which might lead to high local concentrations (66). It remains unknown in which particle fraction ammonia was bound in our samples. A strong binding of ammonia molecules to the inside of dust particles was previously shown so that it is estimated that 40% of total ammonia can be absorbed by dust particles (54, 66). Takai et al. (66) demonstrated that ammonia concentrations were higher in the respirable dust fraction from pig barns compared to the inhalable dust. Ammonia loaded particles probably deposited in the upper respiratory tract, in the tracheobronchial region and in the alveolar region.

The recording production and health data in our study to reveal the positive effect of continuous reduction of ammonia levels during the entire nursery period were time- and cost intensive. The impact of stressors was not clearly reflected by clinical data on this farm. The health status of the pigs was very good during the observation time. There were slight significant differences in the prevalence of skin alterations, coughing and diarrhea between the two experimental groups, which can be deduced to the composition of pig groups brought to the nursery.

As early non-specific inflammatory markers, APP could be useful to measure the success of climate and management improvements on farms. An adequate number of animals should be sampled due to the high inter-individual variance for comparison of data. As shown in this study, the different seasons also meant different burdens on the animals, which are reflected in the level of APP. The hypothesis of the study that adverse effects of dust and ammonia would become obvious in the increased APP also in the absence of clinical signs, could not be confirmed by the results of this study. It proved impossible to show direct influence of ammonia levels on APP concentrations. APP concentrations varied between the different seasons, which were characterized by a couple of the typical climatic challenges described above. The influence of seasonal challenges on CRP- and Hp-concentrations as biomarkers for inflammation as well as a disturbed homeostasis becomes obvious from the results of the two-factorial analysis of variance, although it cannot be confirmed whether increases in APP concentrations in the absence of clinical signs are just physiological adaptation mechanisms. Varying reference values for Hp and CRP based on experimental and field approaches as well as laboratory specific cut-off values exist. In adult boars, CRP reference values were determined to be 3.6–183 μg/mL and significant differences were found between different high health breeding lines (67). In slaughter pigs, Hp concentrations seem to be elevated compared to other published physiological cut-offs (68). In the present study, Hp- and CRP-values were assessed in accordance with the published cut-offs of Heegard et al. (21) because these were evaluated for a non-defined status of inflammation, but also according to age-related reference values (39, 46). Age-related reference values were established in healthy pigs originating from a commercial breeding farm with a high health status. Serum concentrations of CRP, Hp, SAA, and pigMAP generally increase with age with maximum concentrations between week 13 and 16 of age and a significant increase after weaning (39, 46). An increase in Hp- and CRP- concentrations with age was also confirmed in this study. Published concentrations of CRP in 3- and 10-week-old piglets were much lower than those found in this study. Piglets in this study were sampled at the day of weaning, but some hours after separation from the mother and being transported to the nursery compartment due to the duration of the whole experimental procedure on this day. As a rapidly reacting first line APP, CRP concentrations can increase within 4 h after the stimulus (17), so that it cannot be ruled out that acute phase responses had already started. At the beginning of the nursery period, only 5% of the pigs had CRP concentrations lower than those measured in healthy pigs of the same age group in a previous study (39). At the end of the nursery period, all pigs had elevated CRP concentrations. Farm-specific concentrations of APP have been previously described. On a conventional farm, clinically healthy pigs without pathological organ alterations had CRP concentrations exceeding 100 μg/mL (19). CRP concentrations measured in this study were mainly beyond all established cut-offs and might therefore be interpreted as a disturbed homeostasis caused by stressors. Although litters were kept together to avoid mixing stress, the young age of the animals at weaning might be one important stressor on this study farm. At the beginning of the nursery period in the autumn, piglets had higher CRP values, which might also reflect factors already impacting health in the suckling period.

Hp as the most important second line APP can increase later after a stimulus but is elevated for up to 14 days (17). At the end of the nursery period, Hp was higher in the autumn than during the winter, reflecting climate and other stressors during the nursery period. Coughing was more frequent at this time, so that elevated Hp might indicate immune response to biotic or abiotic stimulus in the respiratory tract. The examination of pair-wise samples at the beginning and end of the autumn nursery period for antibodies against most important respiratory tract pathogens revealed that the sow herd was endemically infected without showing clinical symptoms. The decline in antibody levels and number of seroreagents for *A.pp.*, PRRSV, and influenza virus between the day of weaning and end of the nursery period, reflected the decline in maternal antibodies over time without new infections during the nursery period. Taken together, also serological data supported the good health status of the herd.

It can be finally concluded that an APP increase beyond a respective cut-off reflects a disturbed homeostasis in pigs. This can be supported by a study on the prognostic use of Hp concentrations in nursery pigs during week seven of age for future weight gain in fattening pigs (69). High Hp concentrations connected with subsequent low weight gain reflected a status in which more metabolic energy was required to maintain homeostasis. In our study, CRP concentrations at the day of weaning were significantly correlated with the retrospectively calculated FCR (*p* = 0.01), but also with diarrhea (*p* = 0.01) and skin lesions (*p* = 0.05) at the same point of time.

Hp and CRP can be used in combination to assess the health status of swine herds because they have a supplemental diagnostic value (19). As Hp and CRP levels in healthy and diseased pigs overlap on different farms, other than infectious underlying conditions leading to elevated concentrations can trigger the release of Hp and CRP (19).

Surprisingly, already a slight climatic adaptation influenced some production parameters on the study farm, indicating that husbandry improvements might have positive effects not visible during clinical examination. Hp and CRP as non-specific biomarkers for inflammation and stress are appropriate tools for supporting the assessment of health and disease preventive measures on farms.

## Data Availability

The datasets used and/or analyzed during the current study are available from the corresponding author on reasonable request.

## Author Contributions

IH-P conceived the study, the experimental design, and wrote the manuscript. AM performed clinical examinations, evaluations, and sampling on the farm. TB and HS installed all technical devices, performed climate control, and collected climate data. MG and JS developed laboratory methods for dust analysis and supervised all laboratory analyses.

### Conflict of Interest Statement

HS was employed by the company Möller GmbH, Diepholz, Germany. The project was financially and technically supported by Drägerwerk AG & Co. KGaA, Lübeck, Germany. The funder implemented the sensor technique and the technical adaptation of ventilation, but was not involved in analyzing the data or in interpreting the results. The remaining authors declare that the research was conducted in the absence of any commercial or financial relationships that could be construed as a potential conflict of interest.
